# Synergetic Cooperation of microRNAs with Transcription Factors in iPS Cell Generation

**DOI:** 10.1371/journal.pone.0040849

**Published:** 2012-07-13

**Authors:** Jie Chen, Guiying Wang, Chenqi Lu, Xudong Guo, Wujun Hong, Jiuhong Kang, Jianmin Wang

**Affiliations:** 1 Department of Hematology, The Second Military Medical University, Changhai Hospital, Shanghai, People’s Republic of China; 2 Shanghai Key Laboratory of Signaling and Disease Research, School of Life Science and Technology, Tongji University, Clinical and Translational Research Center of Shanghai First Maternity and Infant Health Hospital, Shanghai, People’s Republic of China; 3 Laboratory of Population and Quantitative Genetics, Institute of Biostatistics, School of Life Sciences, Fudan University, Shanghai, People’s Republic of China; Baylor College of Medicine, United States of America

## Abstract

Induced pluripotent stem (iPS) cells were first generated by forced expression of transcription factors (TFs) in fibroblasts. Recently, iPS cells have been generated more rapidly and efficiently using miRNAs with or without other transcription factors. However, the specific and collaborative roles of miRNAs and transcription factors in pluripotency acquisition and maintenance remain to be further investigated. Here, based on the miRNA profiling in mouse embryonic fibroblasts (MEFs), MEFs infected with Oct3/4, Sox2, Klf4 and c-Myc (OSKM) for 1, 2, 4, or 8 day, two iPS cell lines and ES cells, representing iPS activation and maintenance steps, we found that two unique miRNA sets are responsible for different steps of iPS generation, and the miRNA expression profiles of iPS cells are very similar to that of ES cells. Furthermore, we searched for transcription factors binding sites at the promoter regions of up-regulated miRNAs, and found that up-regulated miRNAs such as the miR-429-200 and miR-17 clusters are directly activated by exogenous TFs. The GO and pathway enrichment for candidate target gene sets of miRNAs or OSKM provided a clear picture of division and collaboration between miRNAs and OSKM during completion of the iPS process. Compared with the pathways regulated by OSKM, we found that miRNAs play critical roles in regulating iPS-specific pathways, such as the adherens junction and Wnt signaling pathways. Furthermore, we blocked miRNA expression using Dicer knockdown, and found that the level of miRNAs was decreased following this treatment, and the efficiency of iPS generation was significantly repressed. By combining high-throughput analysis, biostatistical analysis and functional experiments, this study provides new ideas for investigating the important roles of miRNAs, the mechanisms of miRNAs and related signaling pathways, and the potential for many more applications of miRNAs in somatic cell reprogramming.

## Introduction

Mouse embryonic fibroblasts (MEFs) can be successfully reprogrammed to a pluripotent state using four transcription factors (TFs): Oct3/4, Sox2, Klf4 and c-Myc (OSKM), which are identified as reprogramming factors [1]. In 2007, the generation of human induced pluripotent stem cells (iPS) was also reported [2], [3]. As iPS cells exhibit pluripotency and an infinite capacity for self-renewal like embryonic stem cells (ES), and can be established from somatic cells isolated from patients, they are expected to provide new opportunities for disease modeling, the screening of new drugs and personal clinic treatment.

However, reprogramming that results from the induction of defined factors is slow (needs 2 or 3 weeks) and inefficient (less than 1%), suggesting that the four transcription factors are capable, but somewhat insufficient, for cell reprogramming. The slowness and inefficiency of reprogramming may lead to defective reprogramming and not only prevent the clinical applications, but also lead to misunderstandings regarding the mechanisms underlying reprogramming. In addition, the use of proto-oncogenes, such as Klf4 or c-Myc, would increase the risk of tumor formation when integrated into the iPS cell genome. Therefore, many researchers have investigated novel reprogramming factors and/or combinations of these factors, such as L-Myc [4], p53 [5], [6], Tbx3 [7], Glis1 [8], and microRNAs (miRNAs) [9]–[11]. Recent reports have revealed that iPS cells can be generated more rapidly and efficiently by miR-302/367 without any transcription factors, than by OSKM factors [12], indicating a previously known and important role of miRNAs in iPS reprogramming.

In 2008, Marson and his colleagues carried out a systematic analysis of miRNAs and the transcription factors Oct3/4, Sox2, Nanog, and Tcf3 and connected miRNA genes to the core transcriptional regulatory circuitry of embryonic stem cells [13]. It has also been reported that a core developmental signaling network is necessary for pluripotency [14], [15]; however, it remains unclear how these signaling pathways are regulated and whether miRNAs play important roles. Additionally, whether miRNAs or transcription factors play specific and synergistic roles in the pluripotency acquisition during iPS cell generation and maintenance during ES cell passage remains to be investigated further.

Presently, the complex process of iPS generation has been classified into three phases, initiation, maturation and stabilization, based on gene expression profiling and cell morphology changes [16]. It has also been reported that specific miRNAs may play specific roles in the initiation stage [16], [17]; however whether specific miRNAs play critical roles in the acquisition of pluripotency and maintenance of iPS cells remains to be investigated. Here, we found that miRNAs play an important role in the iPS reprogramming process. The iPS process, referred to in this study as the activation and maintenance steps, require corresponding unique sets of miRNAs. GO and pathway enrichment assays for gene sets targeted by miRNAs or OSKM provided a clear picture of specific and synergetic cooperation between miRNAs and TFs, and miRNAs play critical roles in regulating iPS-specific pathways, such as the adherens junction and Wnt signaling pathways. This combination of high-throughput analysis and functional analysis may provide experimental ideas and strategies that are much more effective and facilitate future research on the mechanisms and potentials of miRNAs for application in somatic cell reprogramming.

## Results

### MiRNA Expression Profiling

**Figure 1:**
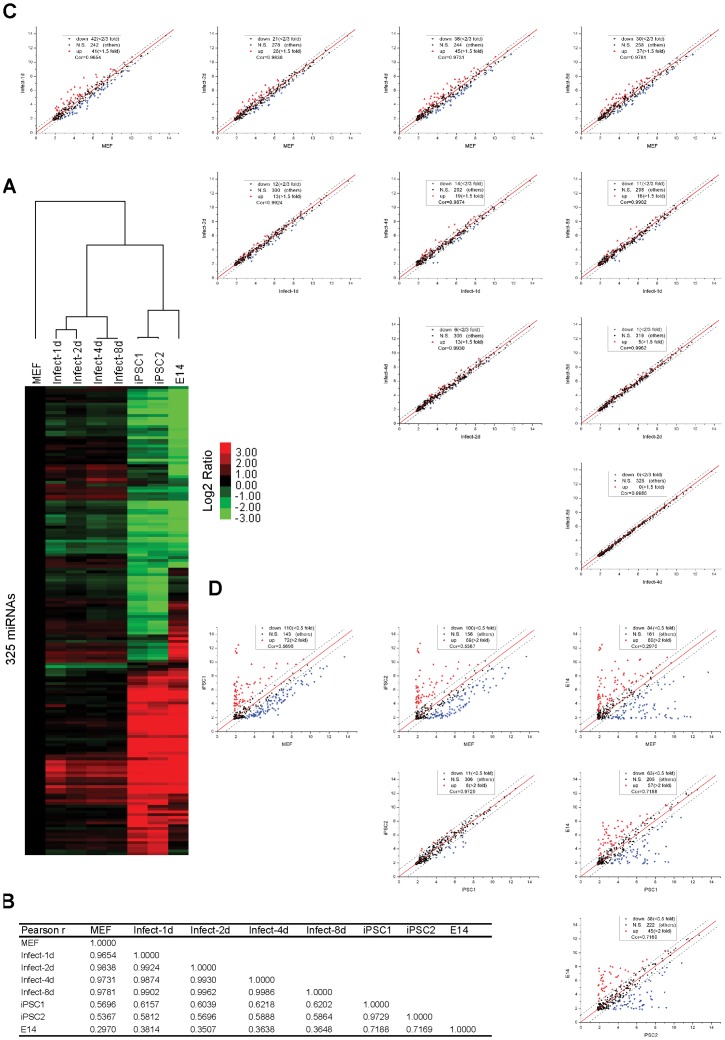
miRNA expression profiling demonstrates differences among MEFs, MEF-infected series, iPS and E14 cells. (**A**) Unsupervised hierarchical clustering of 325 miRNAs (detected in at least one sample) from expression data obtained for mouse fibroblasts (MEFs), the infected series (1, 2, 4 and 8 days post-infection), two iPS cell lines (iPSC1 and iPSC2) and ES cells (E14). Expression values are presented as the log2 ratio of the given miRNA divided by the MEF line. (**B**) The Pearson correlation coefficient matrix between the miRNA expression profiles of all cell lines. (**C**) Pairwise comparisons between MEFs and the infected series (a significant expression signature requires at least a 1.5-fold change in expression (0.585 on the log2 scale)). (**D**) Pairwise comparison between MEFs, iPS and E14 cells (a significant expression signature requires at least a two-fold change in expression (1 on the log2 scale).

Unsupervised hierarchical clustering of miRNA expression data across ES cells, early-infected series, iPS and MEF cells reveals interesting patterns of miRNA expression among these cell types (Figure 1A). Using the methods as shown in Text S1, ES (E14) and iPS (iPSC1, iPSC2) cells have been verified to possess stem cell characteristics, such as being alkaline phosphatase (AP) positive, expressing pluripotency markers, and having the potential to differentiate *in vitro* and *in vivo* (Figure S1). First, even though iPS cells are considered more similar to ES cells than to MEFs, iPSC1 cells are more similar to iPSC2 cells than to ES cells (Figure 1B), which is consistent with previous reports [18], [19]. Second, early-infected series cluster more closely with MEFs than with iPS and ES cells, which in agreement with Pearson correlation analysis (Figure 1B). These results indicate that the iPS process is long and continuous, in which exogenous transcription factors gradually regulate gene (or miRNA) expression patterns similarly to that of ES cells, finally resulting in pluripotency. During the initial days after infection with OSKM (Figure S2), the miRNA expression profile o changes very slightly from day 4 to day 8 (Figure 1C). Then, the profiles of both iPS cells are quite different from OSKM-infected MEF cells but highly similar to that of ES cells (Figure 1B–D). Information for regarding these miRNA expression profiles is presented in Table S1, and quantitative PCR primers of pluripotency markers and exogenous OSKM are listed in Table S2. These results suggest that different sets of genes (or miRNAs) are responsible for different steps of iPS activation and maintenance and that significant change in miRNA levels play important roles in iPS generation.

### MiRNA Expression Signature for Activating the iPS Reprogramming Process

**Figure 2:**
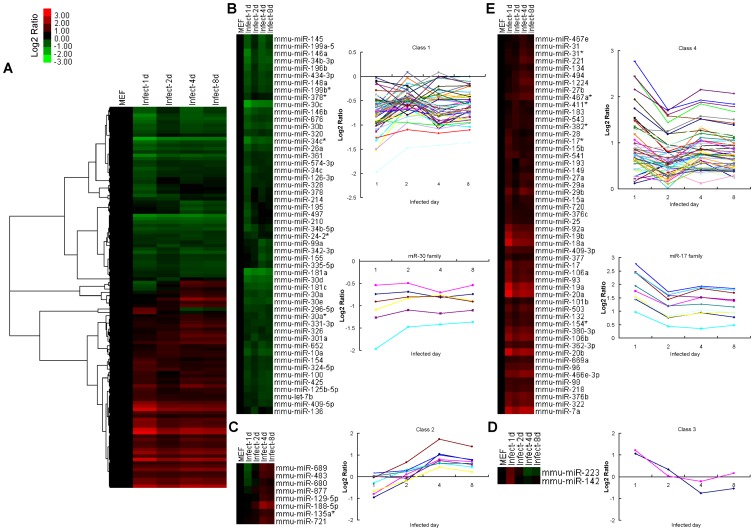
A significant miRNA expression signature for activation of the iPS reprogramming process. (**A**) Unsupervised hierarchical clustering of 114 miRNAs (exhibiting at least a 1.5-fold change in expression between MEFs and the infected series). Expression values are presented as the log2 ratio of the given miRNA divided by the MEF line. (**B**) Fifty-two miRNAs are down-regulated in all cells of the infected series. Two mean signal intensity plots are shown for this group and the miR-30 family. (**C**) Eight miRNAs are down-regulated on the first day and up-regulated on the following days. A mean signal intensity plots is shown for this group. (**D**) Two miRNAs are up-regulated on the first day and down-regulated on following days. A mean signal intensity plots is shown for this group. (**E**) Fifty-two miRNAs are up-regulated in all cells of the infected series. Two mean signal intensity plots are shown for this group and the miR-17 family.

According to a slight change in the early-infected series, a significant expression signature for activating the iPS reprogramming process is that it requires at least a 1.5-fold change in expression between the early-infected series and MEF cells. In total, 114 miRNAs are significantly changed and can be classified into four groups (Figure 2A); 52 miRNAs, including the miR-30 family, are down-regulated during the first 8 days after infection (Figure 2B), 8 miRNAs are down-regulated before day 2 and up-regulated after day 2 after infection (Figure 2C), 2 miRNAs are up-regulated before day 2 and down-regulated after day 2 after infection (Figure 2D), and the remaining 52 miRNAs, including the miR-17 family, are up-regulated (Figure 2E). Based on the miRNA profile of early-infected MEF cells with OSKM, these up-regulated miRNAs such as the miR-17 family may be directly regulated by exogenous OSKM and play important roles in promoting iPS generation.

### MiRNA Expression Signature for the Maintenance of iPS State

**Figure 3:**
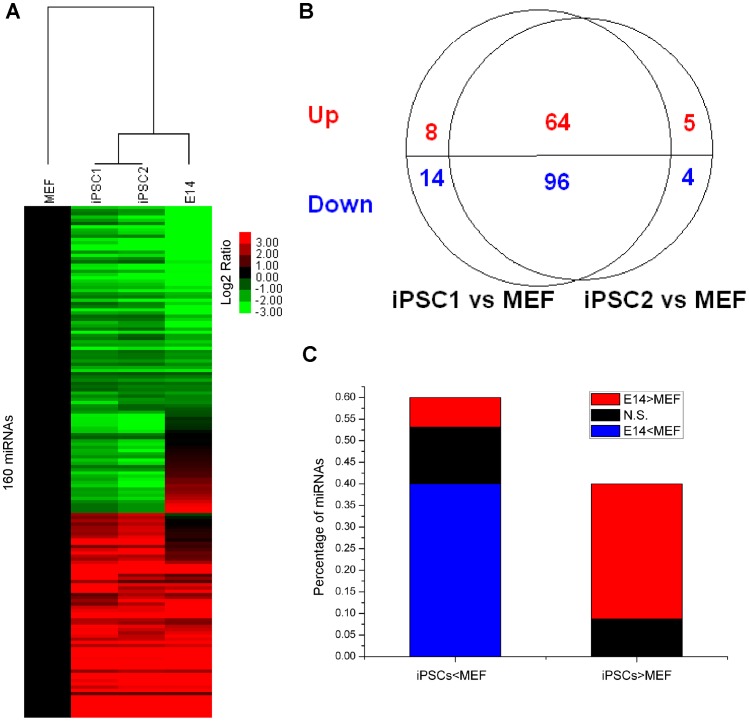
A significant miRNA expression signature for maintenance of the iPS state. (**A**) Unsupervised hierarchical clustering of 160 miRNAs (demonstrating at least a two-fold change in expression among MEFs and both iPS cells). Expression values are presented as the log2 ratio of the given miRNA divided by the MEF line. (**B**) The overlapping pattern of significant miRNA from each iPS cell line (iPSC1, iPSC2) and MEFs (the number of up-regulated miRNAs is shown in red, and the number of down-regulated miRNAs is shown in blue). (**C**) 160 miRNAs were divided into up-regulated (those expressed at higher levels in iPS cells than in MEFs) and down-regulated group. Each group was further sub-classified into three groups: higher expression in ES cells than MEFs (at least a two-fold change in expression) (red), lower expression in ES cells (blue), and the remaining miRNAs (black) (N.S., not significant).

The significant expression signature for maintenance of the iPS state is that it requires at least a two-fold change in expression between iPS and MEF cells. We found that 191 miRNAs that are present at significantly different levels in iPS cells (iPSC1 and iPSC2) compared to MEF cells. The miRNA expression profiles are more similar within iPS cells than between MEF and ES cells (Figure 3A). The expression pattern of iPSC1 is very close to that of iPSC2, and 160 of 191 significant miRNAs are shared by two iPS cells (Figure 3B). Sixty-four miRNAs are up-regulated in both iPS cells compared with MEF cells, and 96 miRNAs are down-regulated. Furthermore, 50 of 64 up-regulated miRNAs and 64 of 96 down-regulated miRNAs are consistent with the trends when comparing ES cells to MEF cells (Figure 3C). Generally, the miRNA expression profiles of iPS cells imitate that of ES cells, which also indicates that iPS cells have ES-like characteristics.

### Common miRNA Expression Signature for the Activation and Maintenance of iPS

Many miRNAs may play important roles in the whole reprogramming process, including the activation and maintenance of pluripotency. We therefore checked the overlapping miRNA expression signature for the activation and maintenance of iPS and found 73 miRNAs which are termed common significant miRNAs. Among these, 34 tend to be down-regulated during the 8 days after infection and in iPS cells, whereas 21 tend to be up-regulated during the 8 days after infection and in iPS cells. The remaining 18 miRNAs exhibit different trends among early-infected MEFs and iPS cells, and these may change significantly from the activation step to the maintenance step.

We then used TAM analyses to determine the enrichment family or cluster of these groups. Among 82 pre-miRNAs for these 73 mature miRNAs, which are commonly involved for activation and maintenance, we found all 7/8 members of the miR-17 family, all 3/3 members of the miR-19 family, 6/6 miRNAs in the cluster of miR-17-92 and 3/3 miRNAs in the cluster of miR-106-93. All of these miRNAs are up-regulated in iPS cells, indicating the importance of miR-17 and miR-19 in the activation and maintenance of iPS pluripotency (Table 1). Among 41 unique miRNA expression signatures for activation of the iPS reprogramming process, we found 4/6 members of the miR-30 family, that are down-regulated. Among 87 unique miRNA expression signatures for maintenance of the iPS state, we found that all 7/7 members of the miR-290 family and all 5/5 members of the miR-8 family are up-regulated; we also found 10/12 members of the let-7 family are down-regulated (Table 2).

**Table 1:** miRNA cluster enrichment analysis of the three miRNA groups.

Group	Cluster	HP	P	HS	S	Exact *P*	Bonferroni	FDR	Members
Common	mir-17-92	48	247	6	6	4.14E-05	1.08E-03	7.90E-04	mir-17;mir-18a;mir-19a;mir-19b-1; mir-20a;mir-92a-1
	mir-25-93-106	48	247	3	3	0.0070	0.18	0.030	mir-106b;mir-93;mir-25
Unique maintenance	mir-290	64	247	7	7	6.08E-05	1.58E-03	7.90E-04	mir-290;mir-291a;mir-291b;mir-292; mir-293;mir-294;mir-295
	let-7	64	247	3	3	0.017	0.44	0.055	let-7a-1;let-7d;let-7f-1
	mir-429-200	64	247	3	3	0.017	0.44	0.055	mir-200a;mir-200b;mir-429
Unique activation	mir-497-195	20	247	2	2	0.0063	0.16	0.030	mir-195;mir-497
	mir-29	20	247	2	2	0.0063	0.16	0.030	mir-29a;mir-29b-1
	mir-30	20	247	2	2	0.0063	0.16	0.030	mir-30c-1;mir-30e

**Table 2:** miRNA family enrichment analysis of the three miRNA groups.

Group	Family	HP	P	HS	S	Exact *P*	Bonferroni	FDR	Members
Common	mir-25	76	422	3	4	0.020	0.73	0.081	mir-25;mir-92a-2;mir-92a-1
	mir-19	76	422	3	3	0.0057	0.21	0.030	mir-19a;mir-19b-2;mir-19b-1
	mir-17	76	422	7	8	3.32E-05	1.23E-03	4.08E-04	mir-106a;mir-106b;mir-17;mir-18a; mir-20a;mir-20b;mir-93
Unique maintenance	mir-290	89	422	7	7	1.53E-05	5.66E-04	2.83E-04	mir-290;mir-291a;mir-291b; mir-292;mir-293;mir-294; mir-295
	mir-8	89	422	5	5	0.00038	0.014	0.0035	mir-141;mir-200a;mir-200b;mir-200c; mir-429
	let-7	89	422	10	12	5.16E-06	1.91E-04	1.91E-04	let-7a-1;let-7a-2;let-7c-1;let-7c-2;let-7d;let-7e;let-7f-1;let-7f-2;let-7g;let-7i
Unique activation	mir-15	44	422	3	5	0.0091	0.34	0.042	mir-195;mir-15a;mir-15b
	mir-29	44	422	3	4	0.0039	0.15	0.024	mir-29a;mir-29b-1;mir-29b-2
	mir-30	44	422	4	6	0.0013	0.049	0.0099	mir-30b;mir-30c-1;mir-30c-2;mir-30e

P represents the number of miRNAs included in all miRNA sets, S represents the number of miRNAs included in miRNA set A, HP represents the number of input miRNAs included in P, and HS represents the number of miRNAs that are of interest included in S; *P* values for all miRNA sets are adjusted using Bonferroni and FDR corrections.

miRNAs with a higher expression level in iPS cells (the infected series) than MEFs are marked in red, whereas miRNAs with a lower expression level in iPS cells are marked in blue.

### Connecting miRNA to the Core Transcription Factors

Based on the miRNA promoter information and TF binding data from Marson et al. 2008, we summarized the transcription factors that bind at the promoter regions of up-regulated miRNAs (Table 3). A strong Oct4 binding signal exists in the promoter region of the mir-17 cluster. We also found a Sox2 binding site in the promoter region of the mir-429-200 cluster and Oct4, Sox2, and Nanog binding sites in the promoter region of the mir-290 cluster. These data clarify why these miRNAs are up-regulated in iPS cells. It is likely that the high level of these miRNA clusters in iPS cells may be directly caused by the ectopic expression of TFs, indicating that the main roles of OSKM in iPS generation may be to up-regulate these miRNAs.

**Table 3:** Transcription factor binding at the promoter regions of up-regulated miRNAs.

Groups	miRNAs	TSS position	Oct4	Sox2	Nanog	Tcf3
Common	mir-17 cluster	chr14:113921300-113927025	Oct4			
	mmir-96-183	chr6:30114875-30130825	Oct4	Sox2	Nanog	Tcf3
	mir-20b-10b/a	chrX:48985925-48991150	Oct4	Sox2	Nanog	Tcf3
Unique activation	mir-31	chr4:88399300-88401975	Oct4	Sox2		
	mir-27a	chr8:87086300-87095525	Oct4	Sox2		
	mir-27b	chr13:63284054-63284254	Oct4	Sox2	Nanog	
	mir-29a-b	chr6:31006975-31008175		Sox2		
	mir-101b	chr19:29167271-29167471		Sox2		
Unique maintenance	mir-290 cluster	chr7:3218001-3219675 [mm9]	Oct4	Sox2	Nanog	
	mir-429-200a/b	chr4:154903109-154903309		Sox2		
	mir-141-200c	chr6:124683151-124685075	Oct4			
	mir-124-1	chr14:63540450-63546275	Oct4		Nanog	
	mir-150	chr7:44988600-44989794	Oct4	Sox2		
	mir-205	chr1:195208350-195211700	Oct4	Sox2	Nanog	
	mir-181d	chr8:87069525-87071900	Oct4	Sox2	Nanog	
	mir-135b	chr1:134018876-134019076	Oct4	Sox2	Nanog	Tcf3
	mir-124-2	chr3:17986635-17986835	Oct4	Sox2	Nanog	Tcf3
	mir-302	chr3:127537000-127537941	Oct4	Sox2	Nanog	Tcf3
	mir-367	chr3:127537000-127537941	Oct4	Sox2	Nanog	Tcf3
	mir-182	chr6:30114875-30130825	Oct4	Sox2	Nanog	Tcf3
	mir-363	chrX:48985925-48991150	Oct4	Sox2	Nanog	Tcf3
	mir-672	chrX:100403736-100403936	Oct4	Sox2	Nanog	Tcf3

miRNA promoter information and TFs binding data were obtained from Table S6 of Marson et al. 2008.

### Comparing the Direct Targets of miRNAs and TFs

We defined direct targets for all significant miRNAs as requiring the agreement of all three popular prediction methods (TargetScan, Pictar and miRnada) and exhibiting a significant opposite tendency (at least a two-fold expression change) in the mRNA expression profiles of iPS cells and MEFs. We found that 527 target genes of 201 miRNAs met these criteria. To classify transcription factors as direct targets, we required a validated TF binding signal and higher expression in iPS cells (at least a two-fold increase in expression relative to MEFs). The mRNA expression data and TF binding information were collected from a previous report [20]. In total, we found 843 transcription factors (OSKM) target genes (Table S3).

**Figure 4:**
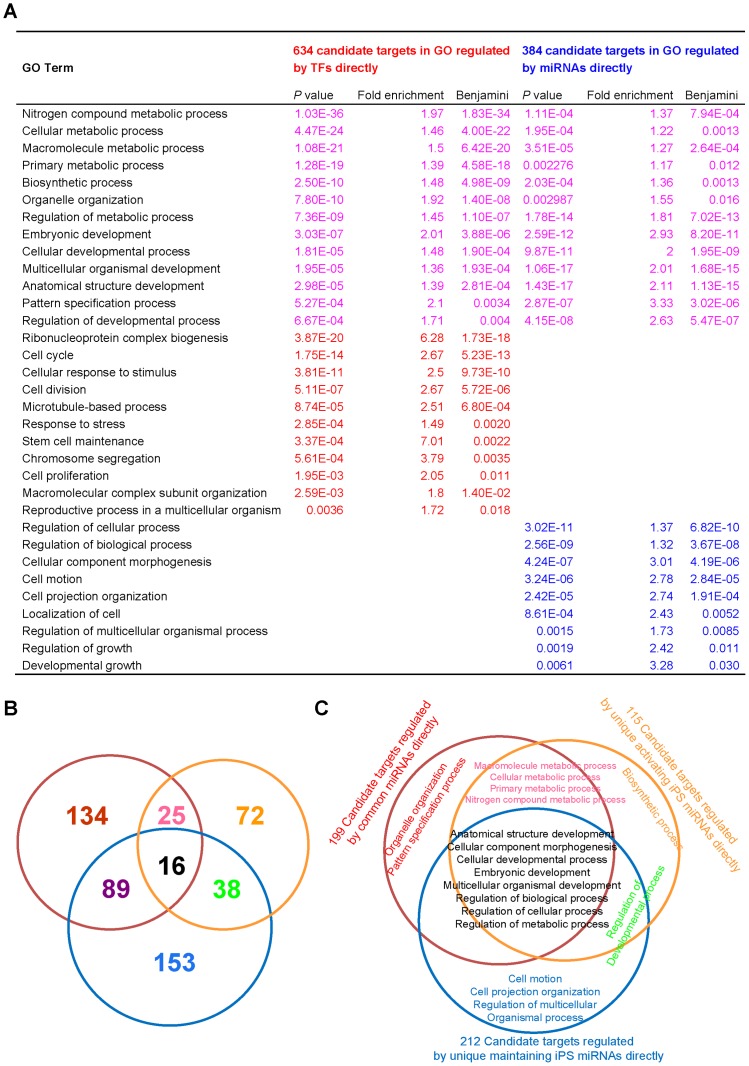
GO enrichment analysis of candidate targets regulated directly by OSKM or miRNA. (**A**) GO enrichment analysis of 843 target genes of 4 TFs (Sox2, Oct4, c-Myc and Klf4). Among these genes, 634 targets are annotated by the GO database (target genes are required to be more highly expressed in iPS cells (at least a two-fold increase in expression relative to MEFs)). GO enrichment analysis of 527 target genes of 201 miRNAs required for the activation or maintenance of iPS; among these genes, 384 targets are annotated by the GO database. (The agreement of all three miRNA-target prediction methods (TargetScan, Pictar, and miRnada) and a significant opposite tendency (at least a two-fold change in expression) in the mRNA expression profiles of iPSCs and MEFs are required for the target genes). (**B**) miRNA target prediction for three groups of miRNAs. Red represents miRNAs shared in common, blue represents unique activation iPS miRNAs, and yellow represents unique maintenance iPS miRNAs. (**C**) GO enrichment analysis for the targets of three miRNA groups (mRNA profiling and TF binding information were obtained from Sridharan et al. 2009).

GO enrichment analyses of the target genes for miRNAs and transcription factors showed that miRNAs and OSKM act both independently and in collaboration to complete the iPS induction process. These two sets of targets focus on metabolic and developmental processes, which are critical for most biological processes, including the iPS process. The direct targets of OSKM are enriched in cell cycle, cell division, cell proliferation, response to stress and stem cell maintenance, whereas the direct targets of miRNAs focus on the regulation of cellular and biological processes, cell motion and cellular component morphogenesis (Figure 4A). Furthermore, we classify the targets of the miRNAs into three directly regulated groups: unique activating iPS miRNAs, unique maintaining iPS miRNAs, and common iPS miRNAs. The overlapping pattern demonstrates a functional division between these three groups (Figure 4B). Using GO enrichment analysis of every section, we found that the targets regulated by common miRNA groups are enriched in metabolism, cellular development, embryonic development and regulation. A particular function of unique activating iPS miRNAs is to regulate biosynthetic targets, whereas unique maintaining iPS miRNAs focus on regulating targets related to cell motion and cell projection organization (Figure 4C).

**Figure 5:**
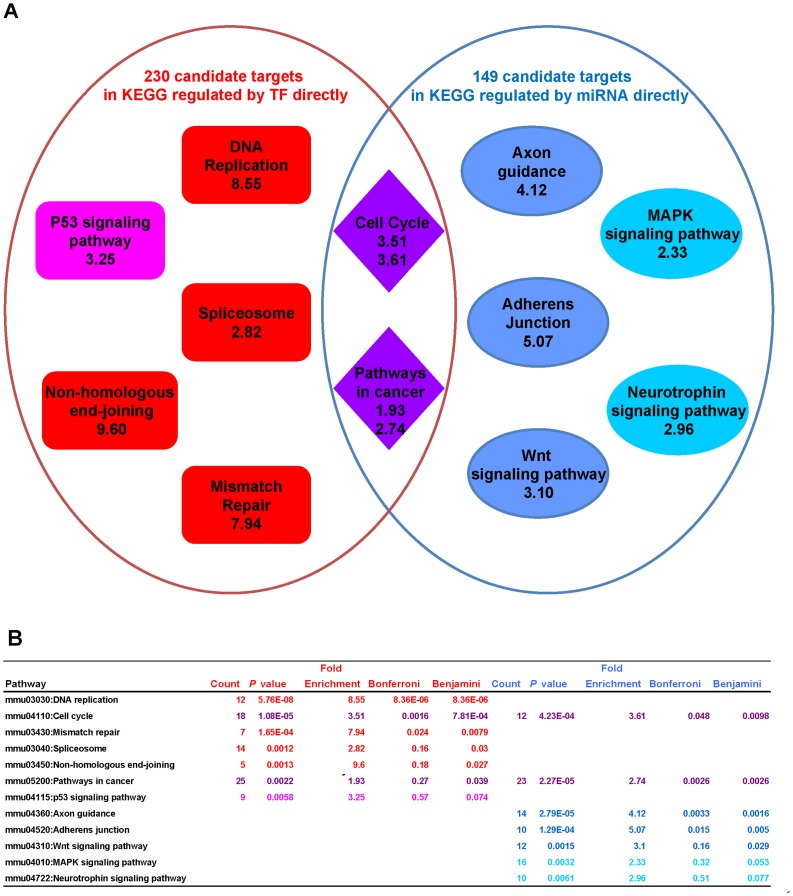
KEGG Pathway enrichment analysis of candidate targets that are directly regulated by OSKM or miRNA. (**A**) Enrichment analysis of 843 target genes of TFs (Sox2, Oct4, c-Myc and Klf4); among these genes, 230 targets are annotated by the KEGG database. Additionally, an enrichment analysis of 527 target genes of 201 miRNAs for the activation or maintenance of iPS; among these genes, 149 targets are annotated by the KEGG database. (**B**) Full information for the pathway enrichment analysis.

**Figure 6:**
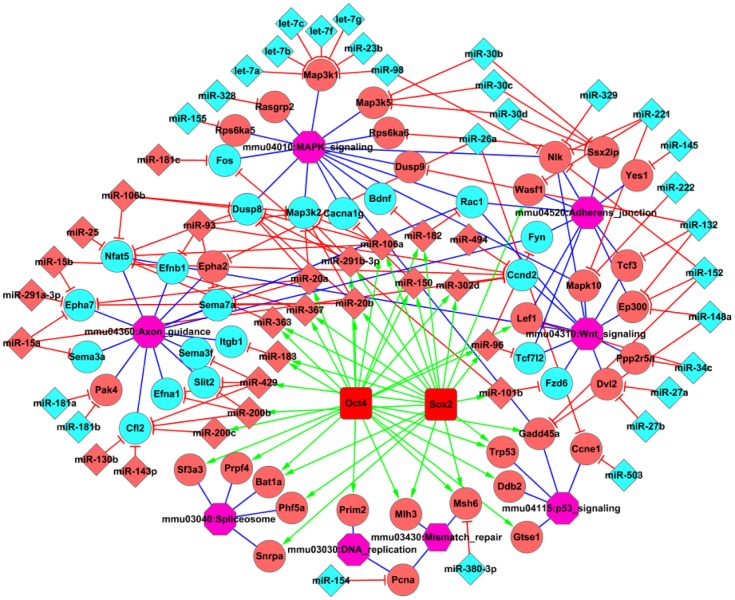
The network for miRNAs, TFs, candidate genes, and signaling pathways resulting from the enrichment analysis. Transcription factors are marked in red squares. Significant miRNAs are labeled using diamond, and candidate target genes of miRNAs and TFs are marked using circles. Pink and bright green colors represent high- and low- expression levels, respectively. Purple octagons represent the related signaling pathways in pluripotency-associated pathways. Green lines represent activation, red lines represent inhibition, and blue lines represent candidate target genes that belong to the corresponding signaling pathways.

Furthermore, pathway enrichment analyses of the two target sets provide a clear picture of division and collaboration in the iPS process between miRNAs and OSKM in regulating signaling pathways (Figure 5A–B). Target genes regulated by OSKM and miRNAs are enriched in the cell cycle and cancer pathways. Furthermore, the direct targets of OSKM are enriched in DNA replication, spliceosome, and mismatch repair, whereas the direct targets of miRNA are enriched in axon guidance, adherens junction and Wnt signaling pathways, which are more specific for iPS generation [15], as shown in a sub-network picture illustrating the relationship between miRNAs, transcriptional factors, predicted target genes, and signaling pathways (Figure 6).

### Critical Roles of miRNAs in the iPS Generation Process

**Figure 7:**
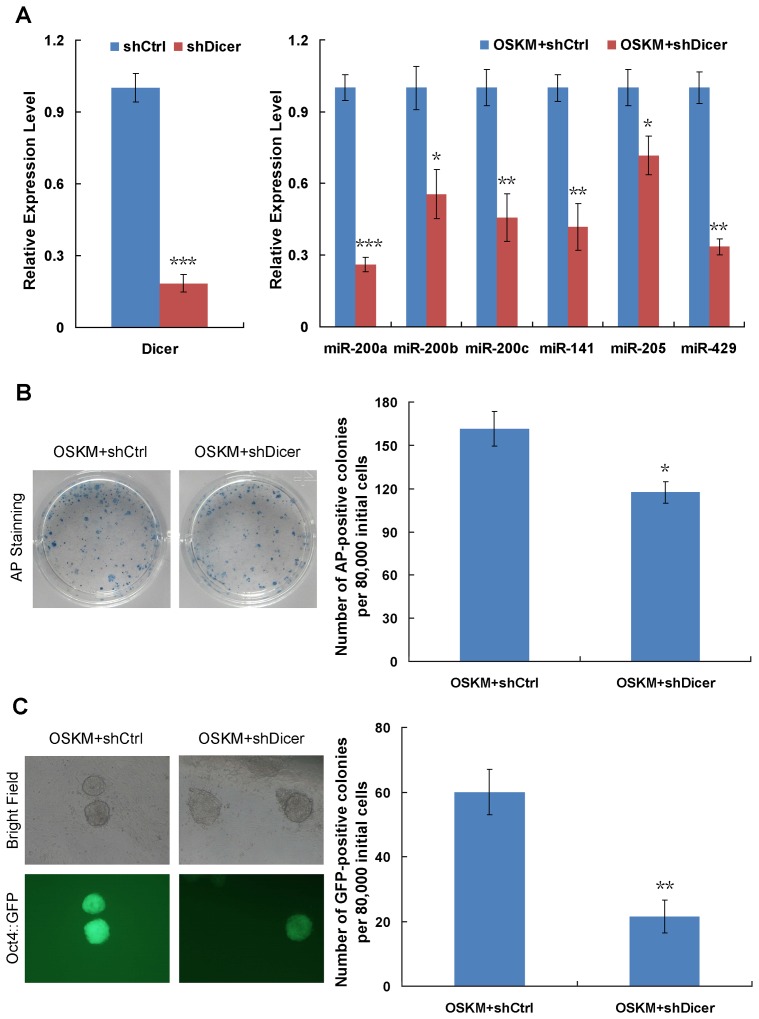
Functional experiments to study the synergetic roles of miRNAs and transcription factors in iPS generation. (**A**) Quantitative analyses of the effects of Dicer knock-down on the expression of miRNAs. GAPDH and U6 are used as internal controls for the gene and miRNA expression assay, respectively. ***P<0.001; **P<0.01; *P<0.05. The error bar represents the standard deviation (SD) of three independent experiments. (**B**) Representative images of AP-positive colonies (left panel) and quantification of AP-positive colony number on day 10 after retroviral infection (right panel). OG-MEFs were infected with retrovirus containing OSKM in combination with scramble shRNA (shCtrl) or Dicer-shRNA (shDicer). Cultures were fixed and stained for alkaline phosphatase activity. *P<0.05. The error bar represents the standard deviation (SD) of three independent experiments. (**C**) Morphology of typical *Oct4*::GFP-positive iPSC colonies and quantification of GFP-positive colony number on day 12 after retroviral infection. Representative phase contrast images (left panel, top) and fluorescence images (left panel, bottom) are shown. As a more stringent quantification of reprogramming efficiency, GFP-positive colony number was counted using a fluorescence microscope (right panel). **P<0.01. The error bar represents the standard deviation (SD) of three independent experiments.

To confirm the synergetic cooperation of miRNAs and transcription factors in iPS generation, we constructed a retroviral vector to knock down the expression of Dicer, a key molecule for the generation of miRNAs. Due to the decreased expression level of Dicer, the expression levels of miRNAs were down-regulated (Figure 7A). When we carried out iPS induction with transcription factors (OSKM) and a knockdown of Dicer, the efficiency of iPS formation was significantly repressed as demonstrated by counting the numbers of *Oct4*::GFP- and AP-positive colonies (Figure 7B–C). These results provide valuable evidence regarding the synergetic roles of miRNAs in cooperation with OSKM during iPS generation.

## Discussion

At present, iPS generation has been classified into three phases, initiation, maturation and stabilization, based on gene profiling during OSKM-induced MEF reprogramming [16]. The initiation phase is elastic, exhibits a characteristic mesenchymal-to-epithelial transition (MET) and mainly reveals changes in cell morphology. Recently, it has been reported that several miRNAs, such as miR-200b and miR-200cc [16], which is activated by p53 [21], and the miR-302-367 cluster [17], which is activated by Oct4/Sox2 [22], [23], can promote the MET process during iPS initiation. However, whether miRNAs are involved in the maturation and stabilization phase and which miRNAs are specifically responsible for the process of iPS generation and pluripotency maintenance remains to be largely investigated. First, we found that different miRNA sets play specific and important roles in the pluripotency activation and maintenance stages of iPS cells based on miRNA profiling.

In the activation step of iPS generation, increased expression of the miR-29 family and decreased expression of the miR-30 family are essential. The combined miRNA expression, miRNA target and signaling pathway assays revealed that the members of the miR-30 family may negatively regulate genes involved in MAPK signaling and adherens junctions [15], whereas the miR-29 family are involved in activating endogenous pluripotent genes such as Oct4 and Nanog by targeting DNMTs [24]–[27]. In addition, high level of the miR-290 and miR-8 families (including the miR-200 clusters) and low levels of the let-7 family play much more important roles in the maintenance steps of pluripotency by regulating axon guidance and MAPK signaling, respectively. Our conclusion was supported by the finding that the embryonic stem cell-specific miR-290 family can promote induced pluripotency [9]. miRNAs, including the previously undiscovered miRNAs involved in reprogramming, targets and related signaling pathways predicted by our present study (Figure 6) may provide useful information for further clarifying the mechanisms of iPS generation.

OSKM transcription factors, in particular Oct3/4 and Sox2 are recognized as the most important reprogramming factors, and to date, viral transduction of OSKM factors remains a robust method to induce iPS cell generation and is often used to fully investigate the mechanisms of pluripotency generation. In our study, we have combined miRNA expression, miRNA targets, TFs binding information, and mRNA expression to study the iPS process, including during the first 8 days post-infection and final iPS cell generation. We found that synergic cooperation between miRNAs and OSKM plays a critical role in completion of the reprogramming process. KEGG pathway enrichment analysis of the candidate targets of miRNAs and OSKM demonstrated that both focus on the cell cycle and pathways involved in cancer. A sub-network based on miRNAs, TFs, candidate target genes, and signaling pathways demonstrated that transcription factors might function through directly binding to the promoter region of specific miRNAs. Interestingly, our study indicated that miRNAs are more specific than OSKM in pluripotency acquirement and maintenance, as indicated by the closer regulation of critical developmental signaling, such as in the adherens junction and Wnt signaling pathways [15].

Somatic cell reprogramming by viral transduction is a double-edged sword. Despite the robustness of the protocol, the host cell viral response acts as a roadblock to efficient reprogramming by initiating a damaging and repressive chain of events, which includes ROS production, DNA damage, the activation of p53 and senescence. Enrichment analysis showed that the direct targets of OSKM are enriched in targets involved in these correlated process or pathways, whereas the direct targets of miRNAs are related to processes and pathways correlated with pluripotency. Rather than replacing one or more transcriptional factors using miRNAs in iPS induction [9]–[11], iPS cells can be generated by using only virus-mediated expression or mature miR-302/367 without the need for any other transcription factors. These miRNA methods were more rapid and efficient than the use of OSKM [12], [28]. A miRNA-based system for selecting and maintaining the pluripotent state in human iPS cells has also been reported recently and represents a straightforward and powerful tool to facilitate the derivation of patient-specific iPS cells [29]. Further study of the specific roles and mechanisms of these signaling pathways in iPS generation and the regulation by candidate miRNAs of developmental signaling pathways will provide further experimental evidence regarding the specific role of miRNAs in iPS cell generation. Obviously, without genetic-integration, these small non-coding miRNAs may have much further potential for application in somatic cell reprogramming.

Aided by high-throughput analyses, such as miRNA profiling analysis, experimental studies can discover not only the phenomena and specific mechanisms behind biological processes but also reveal the entire process, including miRNAs, transcription factors, signaling pathways and the regulatory networks between them. On the basis of high-throughput assays of differently treated samples, we indentified significant miRNA and gene signals using biostatistical analysis. Based on the existing extensive miRNA target database and another study of TFs binding sites, we discovered candidate regulation information for the entire system. The corresponding expression trend in iPS cells and MEFs improved the correctness of these regulatory relationships. We then developed a clear picture of the entire iPS reprogramming process, which involves specificity and collaboration between miRNAs and TFs. This comprehensive analysis of the relationships among candidate molecules, miRNAs, transcription factors, and signaling pathways, may provide more effective experimental strategies and facilitate future research in related fields.

In conclusion, on the basis of the miRNA expression profile during the iPS activation and maintenance stages in MEFs, MEFs infected with OSKM for the first 8 days, iPS and ES cells, we found that unique sets of miRNAs are responsible for various stages of iPS cell generation by combining experimental and biostatistical analysis. Furthermore, GO and pathway enrichment assays provide a clear picture of the synergetic cooperation between miRNAs and TFs. Furthermore, miRNAs play critical roles in regulating iPS-specific pathways, such as the adherens junction and Wnt signalling pathways. These findings may provide new evidence for clarifying the roles and mechanisms of miRNAs and signaling pathways during somatic cell reprogramming.

## Materials and Methods

### Cell Culture

*Oct4*::GFP mouse embryonic fibroblasts (OG-MEFs) were derived from transgenic mice at E13.5 (Takahashi and Yamanaka, 2006). MEF and feeder cells (mitomycin C (Sigma)-treated MEF cells) were cultured in high-glucose DMEM (Hyclone) containing 10% FBS (Biochrom AG) and 1×penicillin/streptomycin (P/S) (Thermo). To maintain Plat-E cells [30], 1 µg/ml of puromycin (Sigma), and 10 µg/ml of blasticidin S (Sigma) were added into high-glucose DMEM (Hyclone) containing 10% FBS (Biochrom) and 1×P/S. Mouse iPSC-1, iPSC-2 [31] and ES cells E14 [32] were maintained in KOSR medium consisting of knockout-DMEM (Gibco) containing 20% knockout serum replacement (KOSR) (Gibco), 1×P/S, 1×nonessential amino acids (NEAA) (Thermo), 1×L-glutamine (Thermo) and β-mercaptoethanol (Gibco) with leukemia inhibitory factor (LIF) (Millipore) on 0.1% gelatin (Sigma)-coated plates with feeder layers. The cells were passaged every two days.

### Vectors

The vectors pMX-Oct4, pMX-Sox2, pMX-Klf4 and pMX-c-Myc were used in this study for four transcription factors [2]. DNA sequence encoding Dicer shRNA (AAGGCTTATCTTCTGCAGGCT) and the scramble shRNA (AATTCTCCGAACGTGTCACGT) were cloned into the retroviral vector pMko.1. All plasmids constructed were confirmed by DNA sequencing.

### Virus Infection and iPS Induction

To generate retroviruses for four transcription factors (OSKM), Plat-E cells were seeded at a density of 8×10^6^ cells per 100 mm dish on the day before transfection. On the next day, pMX-Oct4, Sox2, Klf4 and c-Myc vectors were introduced into the Plat-E cells using Fugene HD transfection reagent (Roche), according to the manufacturer’s instructions. Fugene HD transfection reagent (20 µl) was diluted in 500 µl of Opti-MEM and incubated for 5 min at room temperature (RT). Plasmid DNA (8 µg) was added to the mixture, which was then incubated for 15 min at RT. After incubation, the DNA/Fugene HD mixture was added drop wise onto the Plat-E cells. The cells were then incubated overnight at 37°C under 5% CO_2_. After transfection for 8∼10 h, the medium was replaced with 8 ml of fresh medium, and virus-containing supernatant was harvested at 48 h after transfection. Virus-containing supernatants derived from these Plat-E cultures were filtered through 0.45 µm Millex-HV (Millipore) filters and 4 µg/ml of polybrene (Sigma) was added at a final concentration.

A series of infected-MEF cells was collected after infection with OSKM for 1, 2, 4, and 8 d. For iPS cells induction, OG-MEFs were seeded before infection for 13 h. Viral supernatant was added to the OG-MEFs and centrifuge at 800×g; 90 min later, virus-containing supernatant was replaced with 1 ml of high-glucose DMEM containing 10% FBS (Hyclone) and 1×P/S (Thermo). Two days later, the medium was replaced with KOSR medium containing LIF, β-mercaptoethanol, L-glutamine and NEAA, whereupon the cells were maintained until GFP-positive colonies appeared. *Oct4*::GFP-positive and ES-like colonies were individually mechanically picked up and subsequently maintained on feeders.

### MiRNA Expression Analysis

Mouse miRNA expression data were extracted using an Agilent Mouse miRNA Array. For the analyses, the array data for MEFs, the MEF-infected series, E14 and iPS cells were normalized by using Robust Multichip Analysis (RMA) in R (Bioconductor 2.8). The resulting mouse miRNA data sets contained 325 miRNAs (at least expressed in one sample). Global array clustering was performed using Cluster 3.0 and presented using Java Treeview 1.1.6; miRNA expression values were presented as a log2 ratio compared to MEFs.

### MiRNA Enrichment Analysis

A hypergeometric test was used to determine significant overrepresentation of the miRNA sets among a list of miRNAs of interest. Assuming that P represents the number of miRNAs included in all miRNA sets, S represents the number of miRNAs included in miRNA set A, HP represents the number of input miRNAs included in set P, and HS represents the number of miRNAs that are of interest included in set S, and the probability of HS miRNAs of interest being in miRNA set A is calculated using the TAM method [33]. The P values for all miRNA sets were adjusted by Bonferroni and FDR corrections (R 2.14.0).

### MiRNA Target Prediction

We searched miRNA target information using three popular prediction methods (TargetScan, Pictar, and miRnada) and the miRGen v3 database (http://www.diana.pcbi.upenn.edu/miRGen.html), and the intersection of the data sets was used as a candidate target set.

### GO and Pathway Enrichment Analysis

GO and pathway enrichment analysis of the candidate target sets, which were regulated by miRNAs and TFs are completed using the database DAVID v6.7 for annotation, visualization and integrated discovery [34].

## Supporting Information

Figure S1 **Pluripotency analyses and differential potential of iPSC1, iPSC2 and ES cells.** (**A**) OSKM-derived iPSC1 and iPSC2 colonies exhibit typical stem cell morphology, *Oct4*::GFP positive and high alkaline phosphatase (AP) activity, compared to mouse ES cells (E14). (**B**) QRT-PCR analyses for expression of endogenous pluripotency markers (Oct4, Nanog, Utf1, Dapp5, and Rex1). The error bars represent the standard deviation (SD) of three independent experiments, and GAPDH was used as an internal control. **(C)** OSKM-derived iPSC1 and iPSC2 cells expressed mouse pluripotency markers (Oct4, Nanog, and SSEA-1) similarly to mouse ES cells (E14) by immunostaining. (**D**) Immunostaining shows E14, OSKM-derived iPSC1 and iPSC2 cells can differentiate into cells expressing markers characteristic of the three germ layers, Tuj1 (ectoderm), Gata4 (mesoderm), and HNF-3β (endoderm). (**E**) Teratomas derived from E14, OSKM-derived iPSC1 and iPSC2 cells. Shown are representative images of H&E staining for endoderm (green arrow), mesoderm (red arrow), and ectoderm (blue arrow).
(TIF)

Figure S2 **QRT-PCR analyses for ectopic expression of OSKM in MEFs.** QRT-PCR analyses for expression of exogenous transcription factors Oct4, Sox2, Klf4, and c-Myc in MEFs after infection with OSKM for 1, 2, 4, and 8 days. Relative expression level is presented as the log2 ratio compared to MEFs. The error bars represent the standard deviation (SD) of three independent experiments, and GAPDH was used as an internal control.
(TIF)

Table S1. **Individual miRNA expression data in MEF, MEFs infected with OSKM, two iPS cell lines and ES cells.**
(XLS)

Table S2 **Primer sets used in quantitative PCR assays.**
(DOC)

Table S3 **Genes targeted by transcription factors (OSKM) directly.**
(XLS)

Text S1 **Supplementary methods for quantitative Real-Time PCR, alkaline phosphatase staining and immunostaining,** ***in vitro*** **and** ***in vivo*** **differentiation of iPS cells.**
(DOC)
