# Supplementary figures and images for: Synergetic Cooperation of microRNAs with Transcription Factors in iPS Cell Generation
Source: PLoS One. 2012 Jul 13;7(7):e40849. doi: 10.1371/journal.pone.0040849 (PMC3396613; doi:10.1371/journal.pone.0040849)

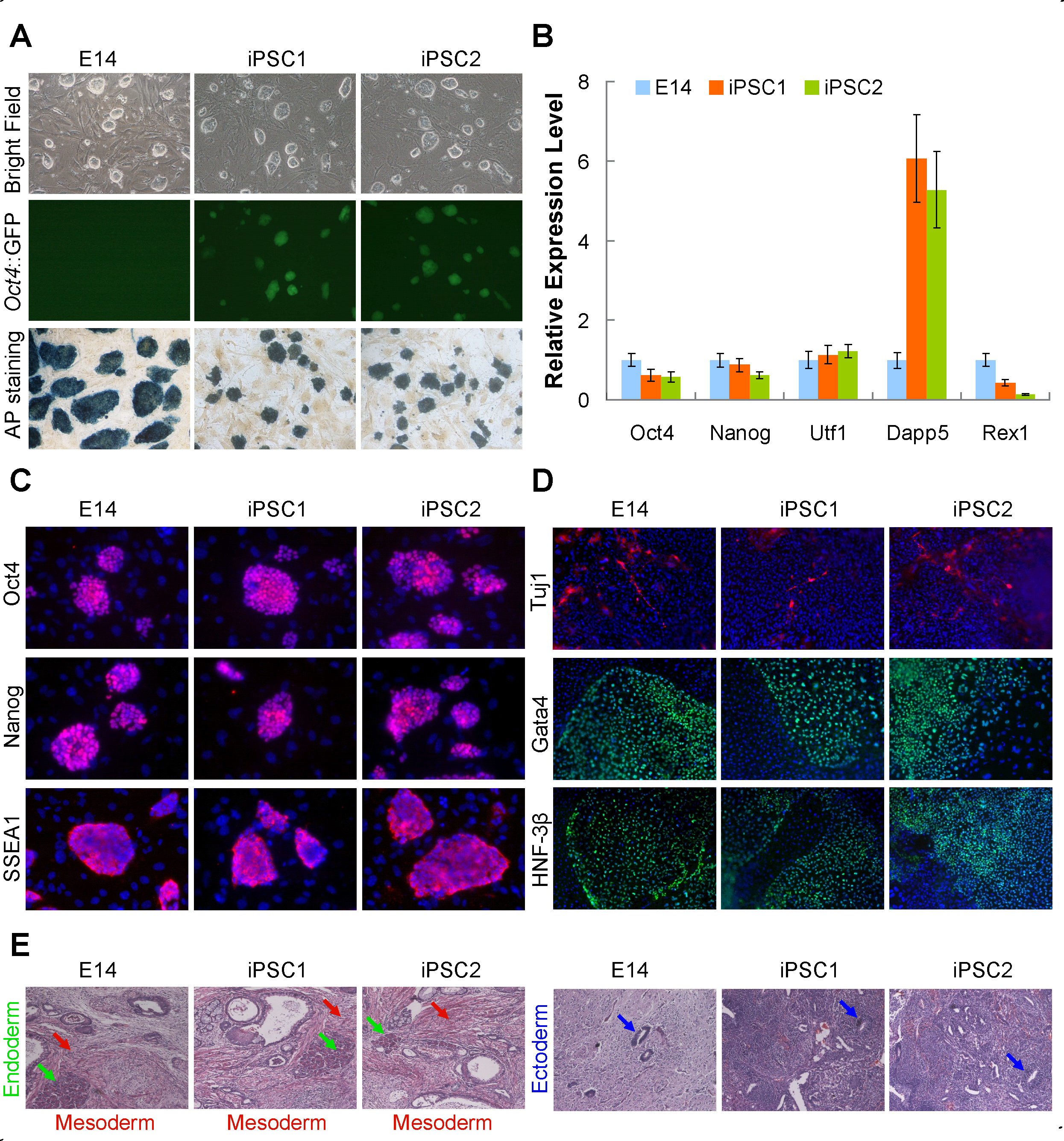

Supplement: Figure S1 — Pluripotency analyses and differential potential of iPSC1, iPSC2 and ES cells. (A) OSKM-derived iPSC1 and iPSC2 colonies exhibit typical stem cell morphology, Oct4::GFP positive and high alkaline phosphatase (AP) activity, compared to mouse ES cells (E14). (B) QRT-PCR analyses for expression of endogenous pluripotency markers (Oct4, Nanog, Utf1, Dapp5, and Rex1). The error bars represent the standard deviation (SD) of three independent experiments, and GAPDH was used as an internal control. (C) OSKM-derived iPSC1 and iPSC2 cells expressed mouse pluripotency markers (Oct4, Nanog, and SSEA-1) similarly to mouse ES cells (E14) by immunostaining. (D) Immunostaining shows E14, OSKM-derived iPSC1 and iPSC2 cells can differentiate into cells expressing markers characteristic of the three germ layers, Tuj1 (ectoderm), Gata4 (mesoderm), and HNF-3β (endoderm). (E) Teratomas derived from E14, OSKM-derived iPSC1 and iPSC2 cells. Shown are representative images of H&E staining for endoderm (green arrow), mesoderm (red arrow), and ectoderm (blue arrow). (TIF) [file pone.0040849.s001.tif]

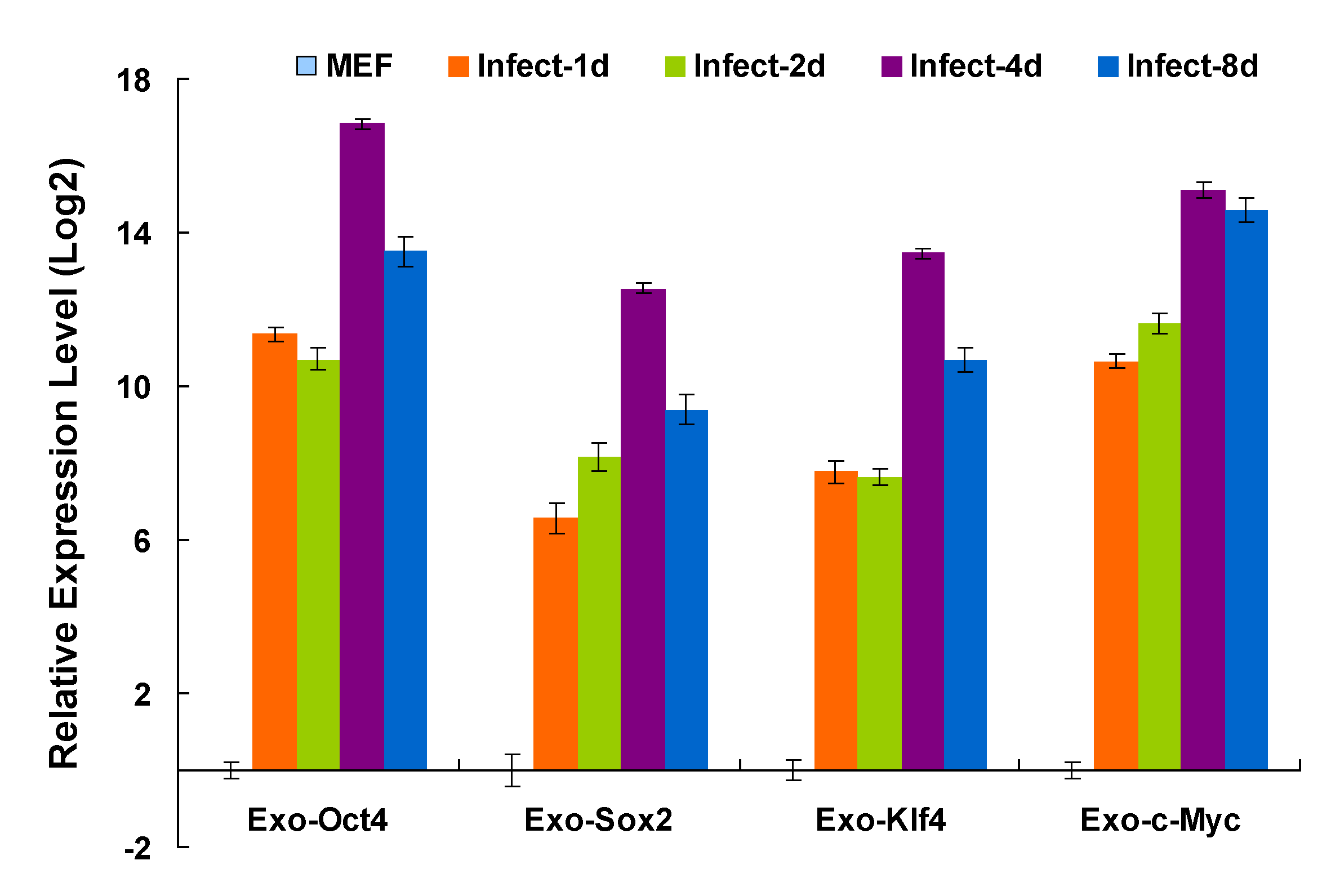

Supplement: Figure S2 — QRT-PCR analyses for ectopic expression of OSKM in MEFs. QRT-PCR analyses for expression of exogenous transcription factors Oct4, Sox2, Klf4, and c-Myc in MEFs after infection with OSKM for 1, 2, 4, and 8 days. Relative expression level is presented as the log2 ratio compared to MEFs. The error bars represent the standard deviation (SD) of three independent experiments, and GAPDH was used as an internal control. (TIF) [file pone.0040849.s002.tif]
