# Supplementary material for: Synergetic Cooperation of microRNAs with Transcription Factors in iPS Cell Generation
Source: PLoS One. 2012 Jul 13;7(7):e40849. doi: 10.1371/journal.pone.0040849 (PMC3396613; doi:10.1371/journal.pone.0040849)
Supplement: Table S2 — Primer sets used in quantitative PCR assays. (DOC) [file pone.0040849.s004.doc]

**Table S2.** Primer sets used in quantitative PCR assays.

| Endo-*Oct4* | TCTTTCCACCAGGCCCCCGGCTC | F |
| --- | --- | --- |
|  | TGCGGGCGGACATGGGGAGATCC | R |
| *Nanog* | AGGGTCTGCTACTGAGATGCTCTG | F |
|  | CAACCACTGGTTTTTCTGCCACCG | R |
| *Dppa5* | ATTCGGGCTAAATGGATGC | F |
|  | TAGCTCCAGGGTCTTCATGG | R |
| *Utf1* | TCTACTGGCCCTGGACG | F |
|  | TGGAAGAACTGAATCTGAGCG | R |
| *Rex1* | ggaagaaatgctgaaggtggagac | F |
|  | agtccccatccccttcaatagc | R |
| *Dicer* | GTCAGCCGTCAGAACTCACTC | F |
|  | ACAGTCAAGGCGACATAGCAA | R |
| *Gapdh* | AGGTCGGTGTGAACGGATTTG | F |
|  | TGTAGACCATGTAGTTGAGGTCA | R |
| pMX-TgUS | GTGGTGGTACGGGAAATCAC | F |
| pMX-Oct3/4-TgDS | TAGCCAGGTTCGAGAATCCA | R |
| pMX-Klf4-TgDS | GGGAAGTCGCTTCATGTGAG | R |
| pMX-Sox2-TgDS | GGTTCTCCTGGGCCATCTTA | R |
| pMX-c-Myc-TgDS | AGCAGCTCGAATTTCTTCCA | R |
