# Supplementary material for: Synergetic Cooperation of microRNAs with Transcription Factors in iPS Cell Generation
Source: PLoS One. 2012 Jul 13;7(7):e40849. doi: 10.1371/journal.pone.0040849 (PMC3396613; doi:10.1371/journal.pone.0040849)
Supplement: Text S1 — Supplementary methods for quantitative Real-Time PCR, alkaline phosphatase staining and immunostaining, in vitro and in vivo differentiation of iPS cells. (DOC) [file pone.0040849.s006.doc]

**Supplementary Methods**

**Quantitative Real-Time PCR for genes and miRNAs expression**

The total RNA was extracted using the TRNzol-A^+^ total RNA Extract Kit (TIANGEN). cDNA synthesis was performed with the TIANScript RT Kit (TIANGEN), following the manufacturer’s instructions. Quantitative PCR analyses were performed in triplicate using the TIANGEN Master Mix (TIANGEN) and Mx3000P QPCR System ([Agilent Technologies](http://www.google.com.hk/url?sa=t&rct=j&q=Stratagene+Mxpro+3000&source=web&cd=3&ved=0CD0QFjAC&url=http%3A%2F%2Fcp.literature.agilent.com%2Flitweb%2Fpdf%2F5989-8240ENUS.pdf&ei=F-fzTsXNNIafiQfX9ZivAQ&usg=AFQjCNG9ZK8sOh3bvLTIQhli6erRC7oL2A)) by normalizing to GAPDH. The primer sequences are listed in Supplementary Table S2. For miRNA quantitative analysis, we used the Bulge-LoopTM miRNA qPCR Primer Set (Ruibobio Co. Cat#MQP-0101) to detect the expression of miRNAs by qRT-PCR assay with U6 as the internal normalized control.

**Alkaline phosphatase staining and immunostaining**

The alkaline phosphatase (AP) staining was carried out using the Vector blue Alkaline Phosphatase Substrate Kit III, according to the manufacturer's protocol. For immunostaining, cells were washed twice with PBS and fixed with 4% paraformaldehyde at room temperature for 20 min. Fixed cells were permeabilized with 0.2% Triton X-100 for 5 min. Cells were then blocked in PBS/10% FBS for 1 h at room temperature. Primary antibody was diluted 1:1000 in PBS/10% FBS. Cells were stained with primary antibody overnight at 4°C, and then washed three times with PBS/10% FBS. Secondary antibody was diluted 1:1000 and cells were stained for 45 min in the dark at room temperature. Cells were stained with anti-Oct4 (Santa Cruz), anti-Nanog (Abcam), anti-SSEA-1 (Santa Cruz) and counterstained with Hoechst33342 prior to detection using fluorescence microscopy to capture both phase and fluorescent images.

***In vitro* and *in vivo* differentiation of iPS cells**

For *in vitro* differentiation of iPSCs, iPSCs were trypsinized into a single cell suspension and the hanging drop method was used to generate embryoid bodies (EBs). For each drop, 1×10^3^ iPSCs in 20 μl KOSR medium without LIF were used. EBs were cultured in hanging drops for three days before being reseeded onto gelatin-coated 48-well plates for another five days. Primary antibodies and dilutions were as follows: anti-Tuj1 (1:500) was obtained from Covance, anti-Gata4 (1:1000), anti-HNF-3β (1:1000) were obtained from Santa Cruz Biotechnology. Cells were counterstained with Hoechst33342 prior to detection using fluorescence microscopy to capture both phase and fluorescent images.

For teratoma formation, 2×10^6^ iPS cells were resuspended with 200 µl DMEM medium and injected into nonobese diabetic/severe combined immunodeficient (NOD-SCID) mice, which are purchased from National Resource Center of Mutant Mice Model Animal Research Center (NARC), NJU. Mice were checked for tumors every week for 4 weeks post-injection. Tumors were harvested and fixed in formaldehyde solution before paraffin embedding, and then for Hematoxylin & Eosin (H&E) staining.
